# Proactive Modulation in the Spatiotemporal Structure of Muscle Synergies Minimizes Reactive Responses in Perturbed Landings

**DOI:** 10.3389/fbioe.2021.761766

**Published:** 2021-12-16

**Authors:** Victor Munoz-Martel, Alessandro Santuz, Sebastian Bohm, Adamantios Arampatzis

**Affiliations:** ^1^ Department of Training and Movement Sciences, Humboldt-Universität zu Berlin, Berlin, Germany; ^2^ Berlin School of Movement Science, Humboldt-Universität zu Berlin, Berlin, Germany

**Keywords:** balance control, modular organization, muscle loading, perturbation-based balance training, motor control, unstable surface training

## Abstract

Stability training in the presence of perturbations is an effective means of increasing muscle strength, improving reactive balance performance, and reducing fall risk. We investigated the effects of perturbations induced by an unstable surface during single-leg landings on the mechanical loading and modular organization of the leg muscles. We hypothesized a modulation of neuromotor control when landing on the unstable surface, resulting in an increase of leg muscle loading. Fourteen healthy adults performed 50 single-leg landings from a 30 cm height onto two ground configurations: stable solid ground (SG) and unstable foam pads (UG). Ground reaction force, joint kinematics, and electromyographic activity of 13 muscles of the landing leg were measured. Resultant joint moments were calculated using inverse dynamics and muscle synergies with their time-dependent (motor primitives) and time-independent (motor modules) components were extracted via non-negative matrix factorization. Three synergies related to the touchdown, weight acceptance, and stabilization phase of landing were found for both SG and UG. When compared with SG, the motor primitive of the touchdown synergy was wider in UG (*p* < 0.001). Furthermore, in UG the contribution of gluteus medius increased (*p* = 0.015) and of gastrocnemius lateralis decreased (*p* < 0.001) in the touchdown synergy. Weight acceptance and stabilization did not show any statistically significant differences between the two landing conditions. The maximum ankle and hip joint moment as well as the rate of ankle, knee, and hip joint moment development were significantly lower (*p* < 0.05) in the UG condition. The spatiotemporal modifications of the touchdown synergy in the UG condition highlight proactive adjustments in the neuromotor control of landings, which preserve reactive adjustments during the weight acceptance and stabilization synergies. Furthermore, the performed proactive control in combination with the viscoelastic properties of the soft surface resulted in a reduction of the mechanical loading in the lower leg muscles. We conclude that the use of unstable surfaces does not necessarily challenge reactive motor control nor increase muscle loading per se. Thus, the characteristics of the unstable surface and the dynamics of the target task must be considered when designing perturbation-based interventions.

## INTRODUCTION

Perturbation-based training interventions are an effective way to improve reactive balance performance and increase muscle strength (Arampatzis et al., 2011; Hamed et al., 2018b; Bohm et al., 2020). Moreover, the effectiveness of perturbation-based interventions for successfully reducing fall risk in different populations has been previously reported (Jöbges et al., 2004; Okubo et al., 2017; Sherrington et al., 2017; Hamed et al., 2018a; Mansfield et al., 2018). Using compliant or unstable surfaces as well as specific treadmill-slips to challenge balance control by introducing external mechanical perturbations (i.e., an alteration of the function of a biological system induced by external mechanism) have been widely used in clinical and training settings (Mansfield et al., 2015; Kurz et al., 2016; Hamed et al., 2018b; Wang et al., 2019). Recently, it was found that exercising mechanisms of dynamic stability control (i.e., increasing the base of support and counter-rotating body segments around the center of mass) in the presence of perturbations improved reactive balance recovery performance and muscle strength already after 3 weeks of exercise in older participants (Bohm et al., 2020). It was proposed that exercising specific balance tasks in the presence of perturbations could increase the demand for the neuromotor system to perceive sensory signals and to generate appropriate motor commands, thus facilitating the sensory-motor integration (Hamed et al., 2018a; Bohm et al., 2020).

External mechanical perturbations increase movement instability (Santuz et al., 2018; Munoz-Martel et al., 2019; Mademli et al., 2021) and challenge the neuromotor system during motion execution. In response, the neuromotor system modifies its strategies to increase control’s robustness (i.e., the ability to cope with perturbations) (Santuz et al., 2018; Munoz-Martel et al., 2019). In earlier studies adopting the muscle synergies approach, we found specific modulations (i.e., wider, less unstable and less complex basic activation patterns of muscle groups) of the temporal structure of muscle synergies in the presence of perturbations (Santuz et al., 2018, 2020; Munoz-Martel et al., 2021). Such regulations of motor function in the presence of perturbations might be related to the efficacy of perturbation-based exercise interventions and its potential to enhance the ability of the motor system to respond and adapt to challenging conditions related to environmental changes during the daily life. Landing-related tasks on unstable surfaces have been widely used in perturbation-based training interventions to induce variable and partly unpredictable disturbances that promotes balance improvement and adaptation (Arampatzis et al., 2011; Hamed et al., 2018a; Bohm et al., 2020). Compliant surfaces have the potential to modify foot kinematics and forefoot stability during landings (Arampatzis et al., 2002, 2005), thus challenging the neuromotor control.

Fundamental basic building blocks defined as motor primitives are compositional elements for movement construction and have been established as kinematic, kinetic, and neural drive entities, which reflect an organizational principle of movement formation (Bizzi et al., 1991; Kargo and Giszter, 2000; Hart and Giszter, 2010; Hogan and Sternad, 2012). It is assumed that a complex movement task can be generated by rearranging and combining motor primitives and therefore motor primitives may provide an insight into underlying neurophysiological mechanisms for motor control (Giszter, 2015). The idea that the neuromotor system faces the redundancy of available degrees of freedom by activating functionally related muscle groups rather than individual muscles is well accepted (Bernstein, 1967; Bizzi et al., 1991). The resultant coordinated patterns of muscle activity are commonly known as muscle synergies and are flexibly combined to produce robust locomotor drive (Mussa-Ivaldi et al., 1994; d’Avella et al., 2003; Bizzi et al., 2008). Synergies—as low dimensional units—produce a complex electromyographic (EMG) pattern in muscles, involving a time-dependent basic activation pattern (temporal structure of the synergy or motor primitives) with variable time-independent weights of activity distribution to different muscles (spatial structure of the synergy or motor modules) (Dominici et al., 2011; Bizzi and Cheung, 2013; Santuz et al., 2017).

Recently, investigating forward and backward lunges on stable and unstable surfaces and using the muscle synergies approach, we found alterations in the spatiotemporal structure of muscle synergies during the stance phase (i.e., weight acceptance and stabilization synergy), resulting in an increased overlap between chronologically adjacent synergies in the unstable condition (Munoz-Martel et al., 2021). However, studies investigating the EMG activity in the lower leg muscles during landings on stable and unstable grounds reported marginal effects of landing surface on the EMG activity (Prieske et al., 2013; Hollville et al., 2020). The biomechanical differences between lunges (movement of the center of mass in both horizontal and vertical direction) and landings (mainly a vertical motion of the center of mass) may affect the effectiveness of proactive neuromuscular adjustments (i.e., before touchdown), resulting in distinct modifications in the spatial and temporal components of the muscle synergies after touchdown in the two tasks. To the best of our knowledge, no study investigated the spatiotemporal activation structure of muscle synergies during landings on unstable surfaces yet. Investigating the spatiotemporal structure of muscle synergies might present an opportunity to better understand the neuromotor control of landings in the presence of perturbations and thus promoting the design of effective exercise programs.

Therefore, the purpose of the current study was to investigate the effects of perturbations induced by an unstable surface on the mechanical loading (i.e., each muscle’s group mechanical demands) and modular organization of neuromotor control during single-leg drop landings. We hypothesized that landing on unstable surfaces would result in a modulation of motor control, reflected in the spatiotemporal components of muscle synergies and in an increase of muscle loading reflected by an increased muscle activity and/or resultant joint moments, in response to the increased challenges in balance control.

## MATERIALS AND METHODS

### Experimental Protocol

We performed an *a priori* power analysis using the findings from our earlier study investigating forward and backward lunges in stable and unstable surfaces (Munoz-Martel et al., 2021). We found an effect size of 1.17 for the differences in the temporal structure of muscle synergies (i.e., width of the motor primitives) between stable and unstable condition, and assuming type I and type II errors of 0.05, we calculated that seven participants were sufficient for the designed study. Fourteen healthy adults volunteered for the study (10 males, 4 females, height 1.75 ± 0.10 m, body mass 67 ± 11 kg, age 28 ± 5 years). None of the participants had a history of acute lower limb injury or back pain in the 6 months preceding the recordings, nor did they suffer from any chronic neuromuscular or musculoskeletal impairments. In accordance with the Declaration of Helsinki, all participants provided written informed consent for the experimental procedure, which was reviewed and approved by the Ethics Committee of the Humboldt-Universität zu Berlin (HU-KSBF-EK_2018_0013).

**FIGURE 1:**
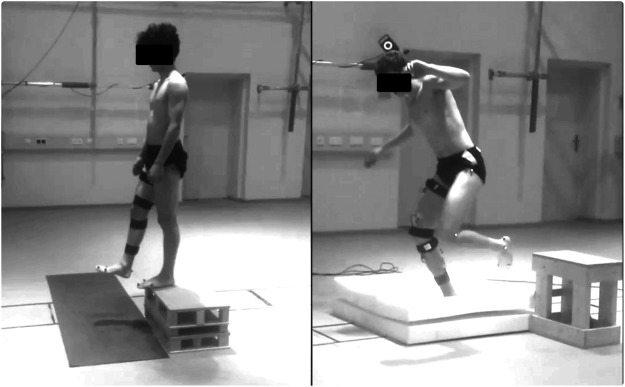
Visual description of the performed task. Participants performed a single-leg landing by dropping onto two ground configurations: stable solid ground **(A)** and two foam pads used as unstable ground **(B)**. Fifty repetitions were performed onto each ground condition and the height of the platform was adjusted to keep a 30 cm distance to the surface.

Participants were instructed to step off a platform, dropping into a single-leg landing (right leg) and maintain the achieved single-leg stance after the touchdown with a strategy of their choice until they felt completely stable (Figure 1). The height of the platform was adjusted to keep a drop height of 30 cm over two possible ground configurations: hard uniform stable ground or unstable ground built out of two 100 × 100 × 10 cm foam pads (one cold foam pad with density = 50 kg/m³ and compressive strength = 6.0 kPa; one polyurethane foam pad with density = 40 kg/m³ and compressive strength = 7.0 kPa). Landings happened over a force plate (40 × 60 cm, AMTI BP400600-200; Advanced Mechanical Technology, Inc. Watertown, MA, USA) sampling the ground reaction force (GRF) at 1 kHz. A minimum of five landings in each condition were used as familiarization and warm-up, after which the participants performed a series of 52 valid landings per condition at a self-managed pace. If the participant was not able to maintain the single-leg stance (e.g., touched the floor with any other part of the body or changed the position of the foot on the ground), the attempt was considered failed and repeated. The order of the series was randomized and a self-managed rest period (minimum 3 min, seating allowed) was given in-between series to avoid fatigue.

A ten-infrared-camera motion capture system (Vicon Motion Systems, Oxford, UK) operating at 250 Hz was used to collect kinematic data from 20 spherical reflective markers (14 mm diameter) placed over the following anatomical landmarks: spinal process of the second, seventh, and 10th thoracic along with the second lumbar vertebrae, and bilaterally over the greater trochanter, lateral and medial epicondyle of the femur, Achilles tendon insertion on the calcaneus, lateral malleolus, tip of the first toe, and the dorsal margin of the first and fifth metatarsal heads. We also assessed the EMG activity of the following 13 right-leg muscles: gluteus medius, gluteus maximus, tensor fasciae latae, rectus femoris, vastus medialis, vastus lateralis, semitendinosus, biceps femoris (long head), tibialis anterior, peroneus longus, gastrocnemius medialis, gastrocnemius lateralis, and soleus using a 16-channel wireless EMG system (Myon m320; Myon AG, Schwarzenberg, Switzerland), with a sampling frequency of 1 kHz. The electrodes were not replaced between series. EMG and force plate analog data streams were collected together with the kinematics and then converted to digital information within the same A/D converter (Vicon MX Giganet).

### Kinetic Analysis

Touchdown of each landing was defined as the first data point of the vertical GRF crossing a 20 N threshold (Malfait et al., 2016). An interval of interest was defined for each landing as the time window between 300 ms prior to the touchdown (flight phase) and until the first point crossing a threshold of body weight ±2.5% following a minimum in the vertical GRF after the touchdown (stance phase). Marker trajectories were filtered using a fourth-order Butterworth low-pass filter with a cut-off frequency of 18 Hz (Malfait et al., 2016). Sagittal kinematics of the ankle, knee, and hip joints from the landing-leg and the resultant internal joint moments for the aforementioned joints were calculated using a custom Matlab (v. R2012a, The MathWorks, Natick, MA, USA) inverse dynamics procedure (Hof, 1992) with segmental masses and inertial parameters derived from literature (Winter 2005). Kinematics and resultant joint moments were time-normalized to 300 points with 100 points assigned to the flight and 200 points to the stance phase, pasted one after another (i.e., concatenated) and kept for further analysis. We calculated the Euclidean norm of the GRF and time-normalized it in the same way as the kinematic and resultant joint moments data. The 2D center of pressure (CoP) data was used to analyze the effect of the ground (SG vs. UG) on the postural sway during the stance phase of each landing. The CoP’s 95% confidence ellipse area (CoP area), representing the area of the smallest ellipse able to contain 95% of all the measured CoP points, was calculated using a custom Matlab script.

### Muscle Synergies

EMG signals were filtered with a fourth-order IIR Butterworth zero-phase high-pass filter with a cut-off frequency of 50 Hz full-wave rectified and low-pass filtered with a cut-off frequency of 20 Hz (Santuz et al., 2017). The amplitude of the EMG signal was then normalized to the maximum activity of each muscle in the SG series of each participant. Lastly, all intervals of interest were time-normalized in the aforementioned manner. Thus, all variables were time-normalized in a similar manner. The rationale for this normalization (i.e., 100 and 200 points to the flight and stance phase, respectively) was to respect the time structure of each landing (i.e., roughly a 1:2 ratio for the flight and the stance) and provide a common time reference for all landings (i.e., the touchdown) while allowing any time-dependent modulation that could have occurred independently of the absolute duration of the events. All EMG off-line processing and further analysis on all variables were performed in R (R v4.0.3, R Core Team, 2020; R Foundation for Statistical Computing, Vienna, Austria).

Muscle synergies were extracted from the filtered and normalized EMG signals and classified using the open source script musclesyneRgies v0.7.1-alpha (Santuz, 2021) based on the classical Gaussian non-negative matrix factorization (NMF) algorithm (Lee and Seung, 1999; Santuz et al., 2017). It is to be mentioned that several other factorization methods have been used in the literature to extract muscle synergies as principal component analysis, independent component analysis, or factor analysis (Tresch et al., 2006; Lambert-Shirzad and Van der Loos, 2017). Nonetheless, NMF has been reported to provide a more intuitive physiological representation of synergies compared with other factorization methods (Lambert-Shirzad and Van der Loos, 2017) and as the best factorization method for identifying muscle synergies in dynamic tasks with different levels of muscle contraction (Rabbi et al., 2020). The concatenated EMG data vectors were grouped in a m × n matrix V, where m = 13 (number of muscles) and n = number of points (300). This matrix was factorized such that V ≈ V_R_ = MP^T^, where V_R_ represents a new reconstructed matrix that approximates the original matrix V, while M and P describe the synergies necessary to accomplish a movement. M represents the m × p motor modules matrix (Gizzi et al., 2011; Santuz et al., 2017), containing the time-invariant muscle weightings. P represents the p × n time-dependent coefficients (motor primitives) matrix (Dominici et al., 2011; Santuz et al., 2017), where p represents the number of synergies necessary to reconstruct the signal and n the number of data points. The number of synergies p was defined as the amount of synergies that did not improve the reconstruction of the signals with the addition of an extra module and it was calculated using the *R* ^2^ between V and VR. When the mean squared error of a linear regression model fitting the curve of *R* ^2^ values versus synergies for all the synergies fell below 10^–5^, we assumed that the addition of an extra synergy did not improve the quality of the reconstruction (Santuz et al., 2017, 2018).

To compare the extracted synergies and give them a functionally meaningful interpretation, we classified them using an unsupervised method based on k-means clustering, with the aim to reduce possible operator-dependent bias in the classification. The algorithm initially clusters the average motor primitives (i.e., one primitive of 300 points per series, average of all the 52 obtained for that series) for each condition separately. This is done for a number of clusters going from one until the number of muscles, with 20 random start sets and using the Hartigan and Wong algorithm (Hartigan and Wong, 1979). Then, a curve “number of clusters vs. within-cluster sum of squares” is built and normalized between zero and one. The minimum number of clusters (or their centroids) is then selected as the number of muscles minus the number of points on the curve that can be linearly interpolated with a mean squared error lower than 10^–3^. Motor modules are then clustered by imposing the number of centroids thus obtained with the analysis on motor primitives. The average full width at half maximum (FWHM) and center of activity (CoA) of the motor primitives are then summed and normalized by the number of points (i.e., 300), and this value is used as a score to compare the k-means classification of modules and primitives. The FWHM was calculated as the number of points exceeding each cycle’s half maximum, after subtracting the cycle’s minimum (Martino et al., 2014), and the CoA is defined as the angle of the vector (in polar coordinates) that points to the center of mass of that circular distribution and its calculation method has been previously described. Common classifiers identify fundamental synergies, while discording classifiers return combined (i.e., spurious) synergies. If no matching is found, only primitive-based classification is retained. Motor primitives between SG and UG condition were compared across condition by means of the FWHM. Furthermore, we calculated the overlapping intervals of the motor primitives for each synergy per every landing trial and then averaged for each participant and surface condition. An overlap is happened when at least two motor primitives were exceeding half maximum at the same time.

To compare motor modules across conditions, we assessed the distribution of muscle contributions for each synergy separately. We defined the ratio of flexor and extensor muscle contribution to each joint in a specific motor module as the coactivation index (CaI). For its calculation, we considered the tensor fasciae latae and rectus femoris as hip flexors and the gluteus medius and gluteus maximus as hip extensors. For the knee, the flexors were the semitendinosus and biceps femoris and the extensors the rectus femoris, vastus medialis, and vastus lateralis. For the ankle, only the tibialis anterior was considered as flexor (i.e., foot dorsiflexor) and the peroneus longus, gastrocnemius medialis, gastrocnemius lateralis, and soleus as extensors (i.e., foot plantar flexors). For each joint, the mean of the flexor contributions Flex and the mean of the extensor contributions Ext were forced to sum to 1:

$$\mathrm{CaI}=\frac{\bar{\mathrm{Flex}}}{\left(\bar{\mathrm{Flex}}+\bar{\mathrm{Ext}}\right)}$$

Hence, the CaI is equal to a) zero when only extensors are contributing to the considered joint, b) one when only flexors are giving their contribution, and c) 0.5 if flexors and extensors are equally contributing (i.e., full coactivation of flexors and extensors).

### Statistical Analysis

After removing the first and last landings, the remaining 50 landings were used to create a representative dataset for each participant on each ground condition of the following variables: FWHM, maximum range of joint angles (defined as the difference between minimum of the joint angle and angle at touchdown), maximum of joint moments and GRF, rate of joint moment development (defined as the ratio between joint moment maxima and the time interval between touchdown and time to maxima), joint moments’ lever arm, and CoP area. Then the mean of the 50 repetitions of each participant was used as the participant’s data for the statistical test. We tested the homogeneity of variances on the residuals of each aforementioned variable using Levene’s test. If the variables were normally distributed, we used a parametric test to investigate the effect of ground condition on variable. Hence, we performed a one-way ANOVA for repeated measures on each of the following variables: GRF maxima, CoP area, and FWHM of the synergies. Correspondingly, we used a two-way ANOVA for repeated measures on the joint kinematics, resultant moments, joint moment’s lever arm, and joint moment’s rate using ground (SG–UG) and variable (i.e., ankle, knee hip joint angle or moment) as within-subjects variables. The same two-way ANOVA for repeated measures was used for each synergy using ground (SG–UG) and muscle or CaI, for the motor modules as within-subjects variables. When normality conditions on the residuals were not met (i.e., joint range of motion, resultant joint moment maxima, and FWHM of the touchdown synergy), we used a rank-based robust ANOVA from the R package “Rfit” (v 0.24.2, function “raov”) (Kloke and McKean, 2012). If an interaction of main effects was observed, we performed a Tukey *post hoc* analysis with false discovery rate α-value adjustment. All the significance levels were set at 0.05.

Moreover, we adopted a similar approach using the statistical parametric mapping (SPM) on all the aforementioned continuous variables (i.e., time-normalized vectors). Correspondingly, the individual time-normalized joint kinematics, resultant joint moments, GRF, EMG, and overlaps curve for each landing were averaged to create a representative dataset of each participant on each ground condition. We tested for normality using a D’Agostino–Pearson test corrected for arbitrary one-dimensional domains using random field theory (Pataky, 2012). If non-parametric tests were needed, the corresponding two-way ANOVA for repeated measures permutation test (Nichols and Holmes, 2002) was used. SPM allows us to analyze the entire time series by using random field theory (Naouma and Pataky, 2019). Based on the temporal smoothness of the data (i.e., each time-normalized dataset) residuals trajectory, a critical threshold f* was calculated. Then a test statistics SPM{F} was evaluated at each point of the time series. In the case that SPM{F} exceeded f*, a significant difference was detected. Similar to the previously described analyses, significance level was set at 0.05. In case of finding an interaction of main effects, we conducted a SPM two-tailed paired *t*-test with significance t* level Bonferroni corrected for multiple comparisons (n = number of levels in the variable) between each relevant pair of variables as a *post hoc* analysis. All SPM calculations were performed using the open-source package spm1d (v 0.4.3).

## RESULTS

**FIGURE 2:**
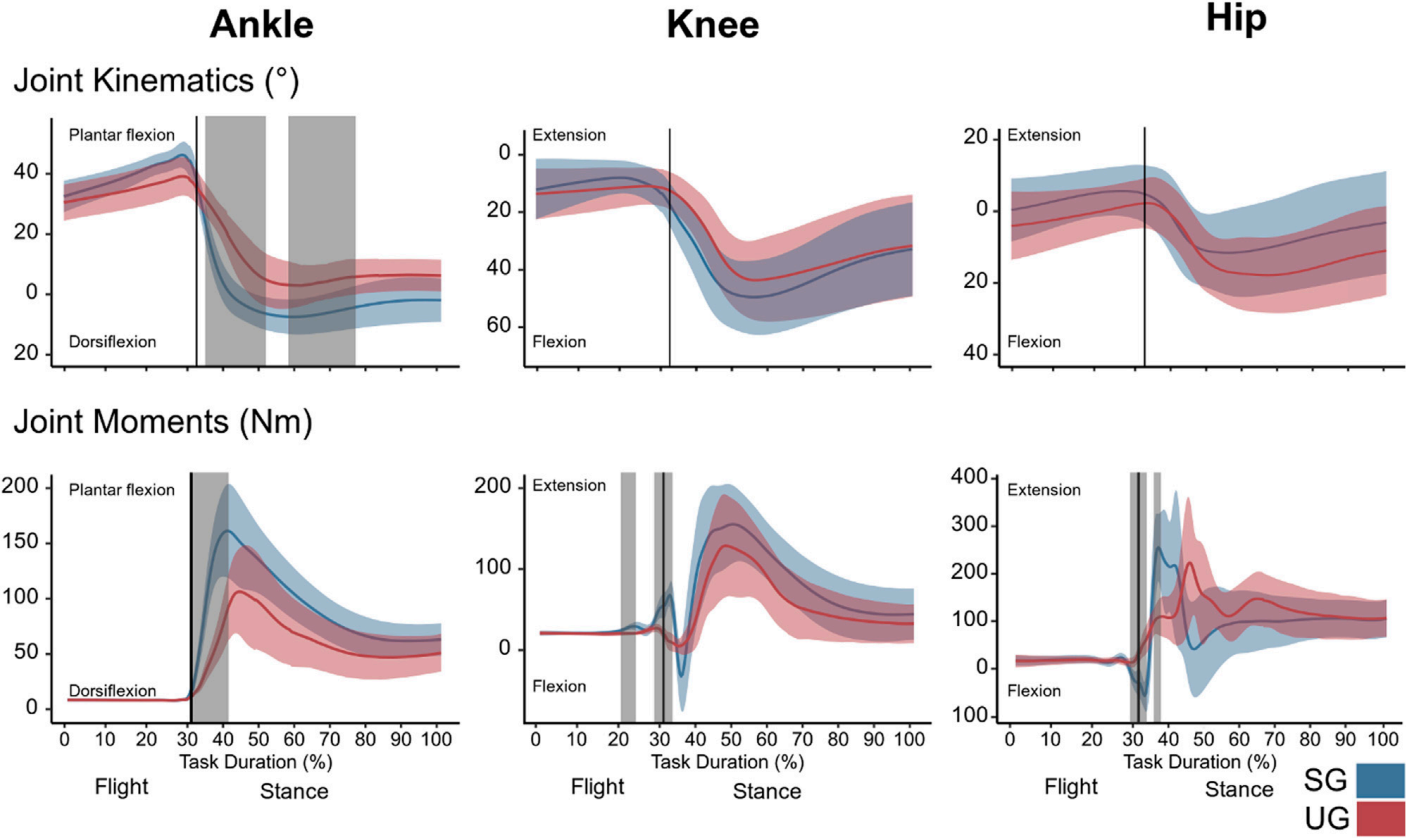
Lower limb kinematics and internal resultant joint moments of the single-leg drop landing (from 300 ms previous to the touchdown until the first point crossing a threshold of body weight ±2.5% following a minimum in the vertical ground reaction force after the touchdown). Each panel shows the mean values and SD bands for the ankle, knee, and hip joint angles and moments for the stable (SG—blue) and unstable (UG—red) ground condition. Panels are presented in a time-normalized base; vertical lines represent the touchdown. Gray vertical bands highlight time periods of significant differences assessed by statistical parametric mapping.

Participants needed a longer time to reach their body weight threshold (i.e., stabilization) when landing on UG. This led to a significantly longer stance phase after landing onto the unstable ground compared with the stable condition (SG: 0.491 ± 0.062 s, UG: 0.629 ± 0.085 s, t(1,13) = −5.611, *p* < 0.001). Two participants were excluded from the kinematic analysis due to poor reconstruction of the markers’ trajectories. The SPM analysis revealed a significant main effect of the ground type on joint kinematics during the flight (F* = 9.877, *p* = 0.012) and the first half of the stance phase (F* = 9.877, *p* = 0.034). An interaction of ground by joint was found shortly before touchdown and during the entire stance phase (F* = 5.724, *p* < 0.001). The *post hoc* analysis revealed no differences in the flight phase in a specific joint but showed that landing on UG led the participants to reach a less dorsiflexed position at the ankle joint after the touchdown (35–55% of the task duration, t* = 3.618, *p* = 0.010) and in the middle of the stance phase (59–78% of the task duration, t* = 3.618, *p* = 0.007, Figure 2). Landing on UG also had a significant main effect on the joint range of motion (F(1,11) = 5.48, *p* = 0.023) and a significant interaction of ground by joint (F(2, 22) = 9.81, *p* < 0.001). The *post hoc* analysis showed that landing on UG resulted in a less range of dorsiflexion at the ankle joint during the stance phase (UG: 37.19 ± 12.76°, SG: 52.03 ± 6.45°, *p* < 0.001).

**FIGURE 3:**
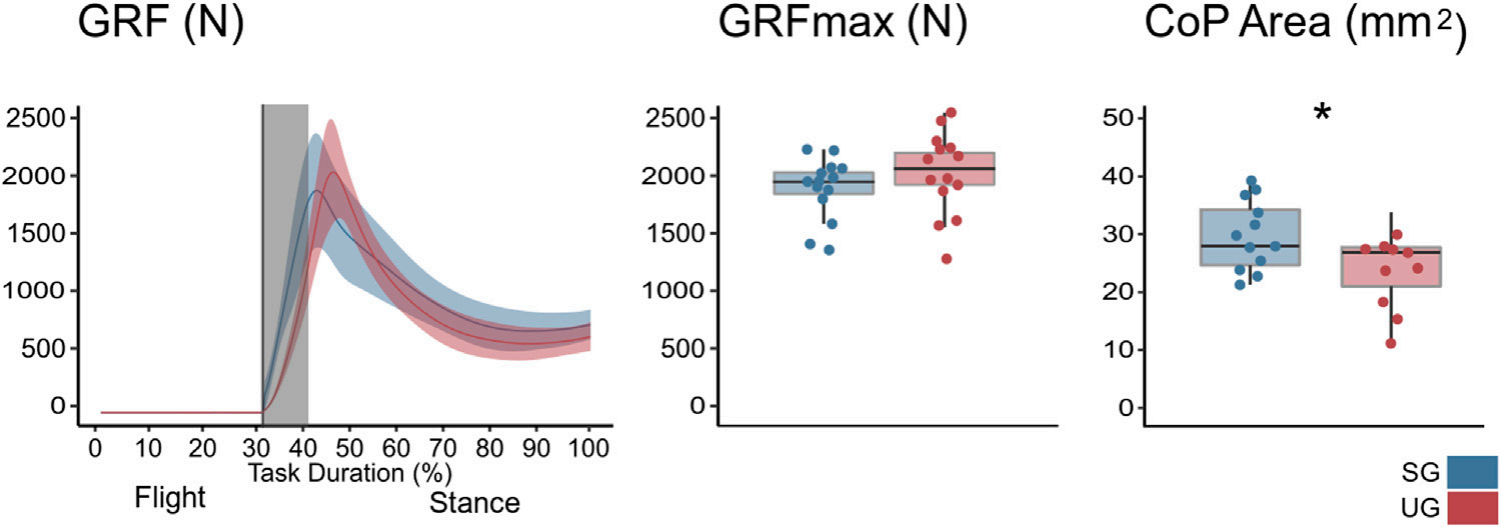
Right panel presents the mean Euclidean norm and SD bands of the ground reaction force (GRF) during a single-leg drop landing for the stable (SG) and unstable ground conditions (UG). Vertical lines represent touchdown; gray vertical bands highlight time periods of significant differences assessed by statistical parametric mapping. Central panel represent the maximum of the GRF with points denoting single trials. Left panel shows the CoP 95% confidence area for the stance phase with points denote single trials. Asterisks denote statistically significant (*p* < 0.05) differences between the two conditions.

There was a significant ground effect on the internal resultant joint moments shortly after the touchdown (F* = 17.500, *p* = 0.003) and an interaction of ground by joint in the swing phase (∼20–25% of the task duration, F* = 8.572, *p* = 0.025), around touchdown (F* = 8.572, *p* = 0.012), and during three periods of the stance phase: between 36 and 40% (F* = 8.572, *p* = 0.023), 45–75% (F* = 8.572, *p* < 0.001), and between 60 and 76% of the task duration (F* = 8.572, *p* < 0.001). The *post hoc* analysis showed a lower plantar flexion moment at the ankle joint (∼30–40% of the task duration, t* = 4.097, *p* = 0.002) in UG compared with SG (Figure 2). At the knee joint, the extension moment was also lower in UG during the flight phase (20–25% of the task duration, t* = 4.118, *p* = 0.017) and around the touchdown (28–34% of the task duration, *p* = 0.010, Figure 2). A lower hip flexion moment in UG condition around the touchdown (∼30% on the task duration, t* = 4.166, *p* = 0.008) was found (Figure 2). Furthermore, the maximum resultant ankle (*p* = 0.002) and hip (*p* = 0.004) joint moment and the rate of moment development in all joints (*p* = 0.029 at the knee and *p* < 0.001 for the ankle and hip) were significantly lower in UG compared with SG (Table 1). The lever arm of ankle joint center to GRF vector at moment maximum was also lower in the UG condition (Table 1). The SPM analysis identified a significantly lower GRF after touchdown in UG (t* = 3.305, *p* = 0.013, Figure 3); however, the maximum of the GRF did not differ (F(1,13) = 2.025, *p* = 0.178, Figure 3) between the two ground conditions. CoP area during the landing was smaller in UG (F(1,14) = 7.527, *p* = 0.020) compared with SG (Figure 3).

**TABLE 1:** Maxima of the resultant joint moment, lever arm at moment maxima, and rate of moment development for the ankle, knee, and hip joint during a single-leg drop landing on stable (SG) and unstable ground (UG). Values are presented as mean ± SD. Asterisks denote statistically significant (*p* < 0.05) difference between the two ground conditions.

Joint	Parameter	SG	UG
Ankle	Moment max (Nm)*	183.7 ± 46.5	142.4 ± 41.4
	Lever arm (m)*	0.102 ± 0.02	0.079 ± 0.02
	Rate of moment (Nm/s)*	2,665 ± 487	1,094 ± 275
	Time to peak torque (ms)	56 ± 14	60 ± 21
Knee	Moment max (Nm)	136.6 ± 41.1	118.5 ± 53.2
	Lever arm (m)	0.093 ± 0.02	0.077 ± 0.03
	Rate of moment (Nm/s)*	1,593 ± 554	788 ± 260
	Time to peak torque (ms)	73 ± 11	86 ± 22
Hip	Moment max (Nm)*	261.6 ± 78.1	207.5 ± 92.1
	Lever arm (m)	0.104 ± 0.02	0.090 ± 0.01
	Rate of moment (Nm/s)*	4,514 ± 1923	1,610 ± 806
	Time to peak torque (ms)	77 ± 13	78 ± 23

**FIGURE 4:**
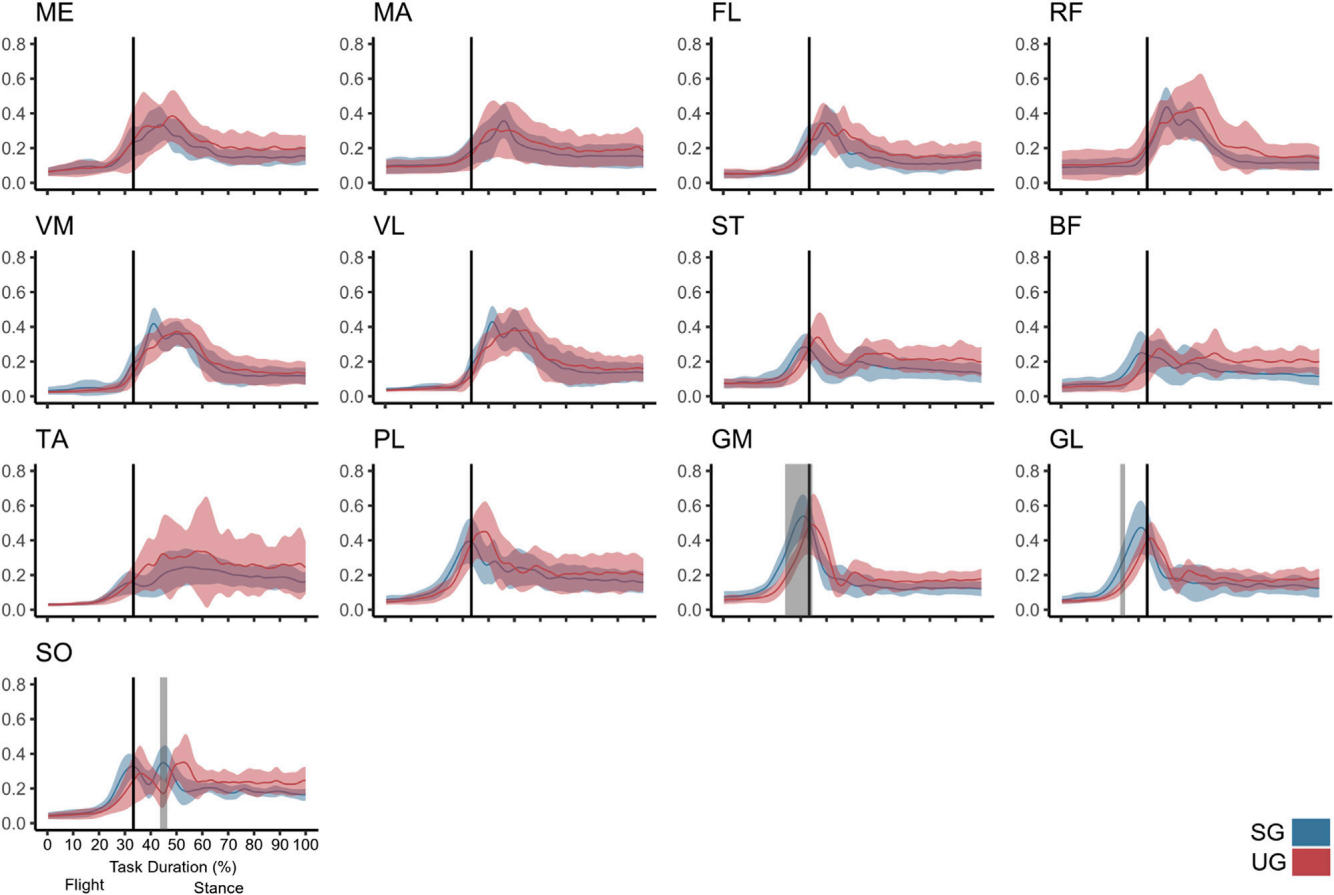
Mean values and SD bands for the EMG activities for a single-leg drop landing on stable (SG, blue) and unstable ground condition (UG, red) normalized to the maximum activity of each muscle on the SG condition. Vertical lines represent touchdown. Gray bands denote time periods of significant difference found by the statistical parametric mapping analysis. ME, gluteus medius; MA, gluteus maximus; FL, tensor fascia latae; RF, rectus femoris; VM, vastus medialis; VL, vastus lateralis; ST, semitendinosus; BF, biceps femoris (long head); TA, tibialis anterior; PL, peroneus longus; GM, gastrocnemius medialis; GL, gastrocnemius lateralis; SO, soleus.

The ground condition affected the EMG activity during the second half of the swing (F* = 14.364, *p* < 0.001), and in three brief periods of the stance phase (*p* = 0.049, 0.014, and 0.029). There was also a significant interaction between ground and muscle in both the flight (F* = 2.718, *p* < 0.001) and stance (F* = 2.718, *p* = 0.001) phase. The *post hoc* analysis revealed lower EMG activity before touchdown in the gastrocnemius medialis (∼25–33% of the task duration, t* = 4.544, *p* < 0.001) and gastrocnemius lateralis (∼25% of the task duration, t* = 4.447, *p* = 0.004) and after the touchdown in the soleus (∼45% of the task duration, t* = 4.709, *p* = 0.020, Figure 4) in the UG condition.

**FIGURE 5:**
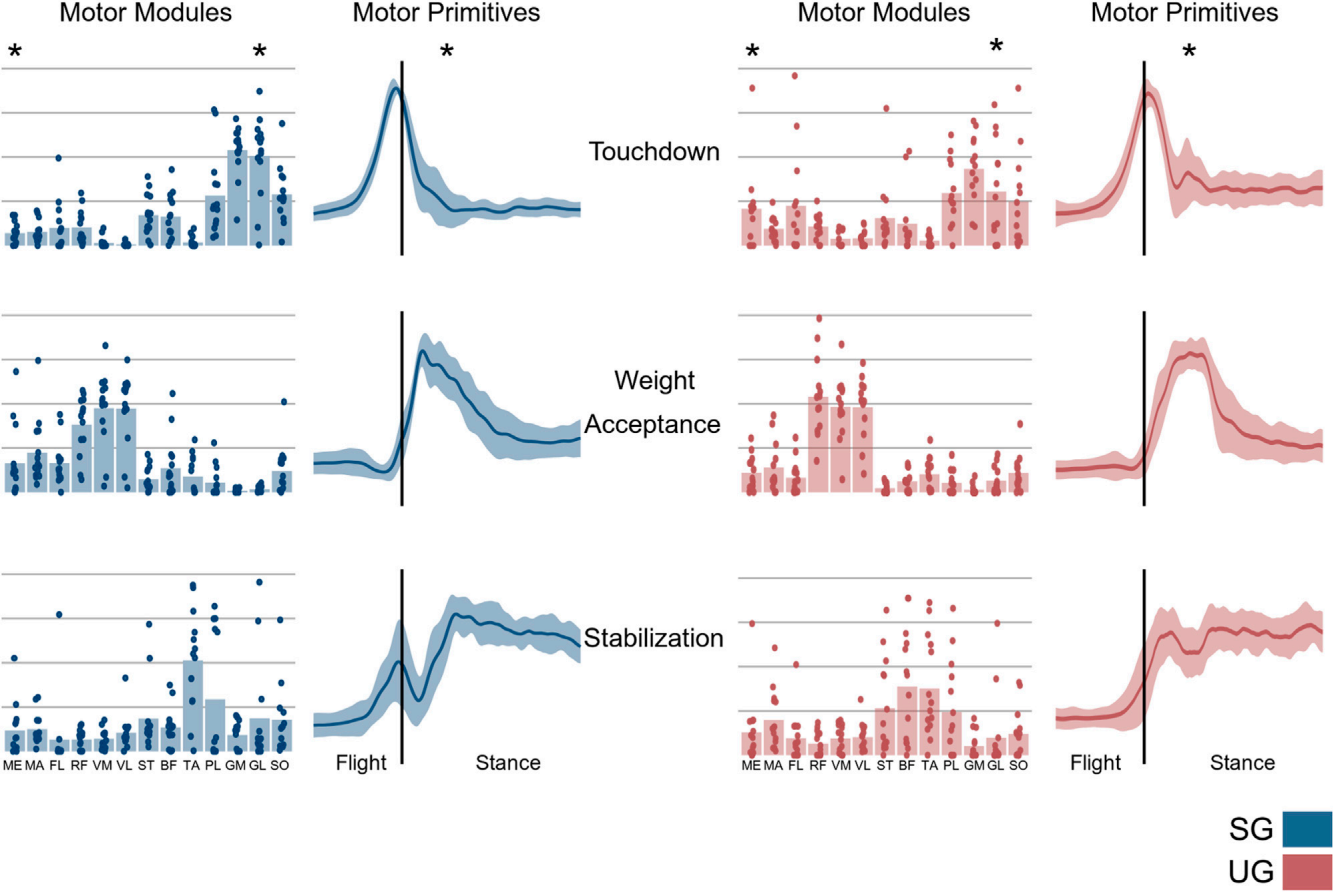
Average and individual motor module values and average with their SD bands motor primitives of the fundamental synergies classified from a single-leg drop landing on stable (SG, blue) and unstable (UG, red) ground. The vertical lines in the primitive panels indicate the touchdown. ME, gluteus medius; MA, gluteus maximus; FL, tensor fascia latae; RF, rectus femoris; VM, vastus medialis; VL, vastus lateralis; ST, semitendinosus; BF, biceps femoris (long head); TA, tibialis anterior; PL, peroneus longus; GM, gastrocnemius medialis; GL, gastrocnemius lateralis; SO, soleus. Asterisks denote *post hoc* (*p* < 0.05) differences in the motor modules and width on the motor primitives between stable (SG) and unstable (UG) condition.

**FIGURE 6:**
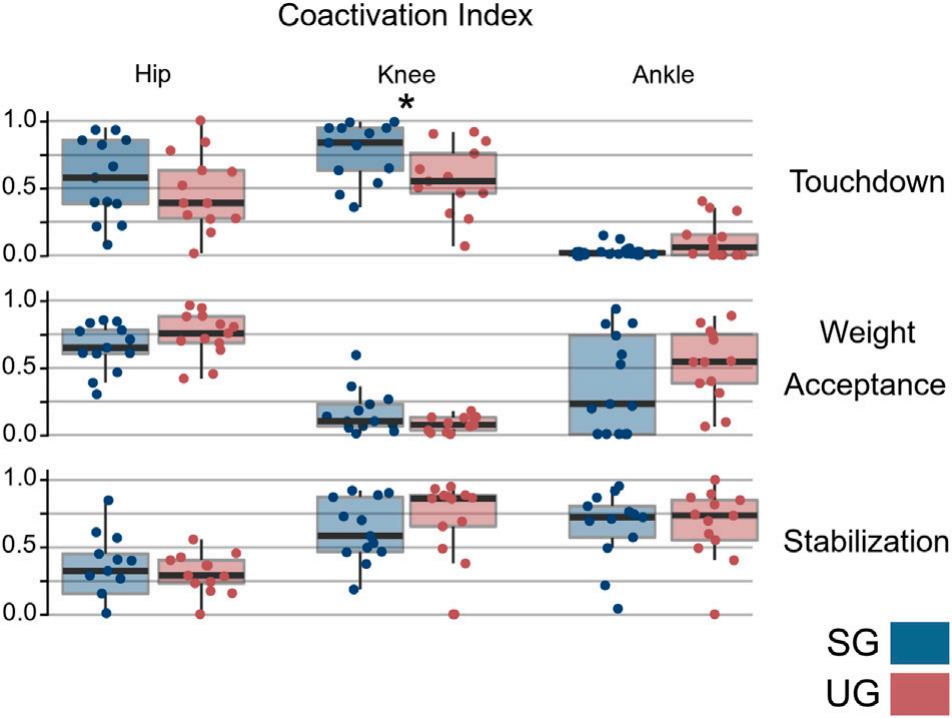
Coactivation index (CaI) for the motor modules for the recognized synergies. The CaI may vary from 0 (exclusive contribution of extensors) to 1 (exclusive contribution of flexors). A CaI of 0.5 indicates equal contribution of flexors and extensors for that motor module. Points denote single trials and asterisks denote statistically significant (*p* < 0.05) differences between stable (SG) and unstable (UG) conditions.

**FIGURE 7:**
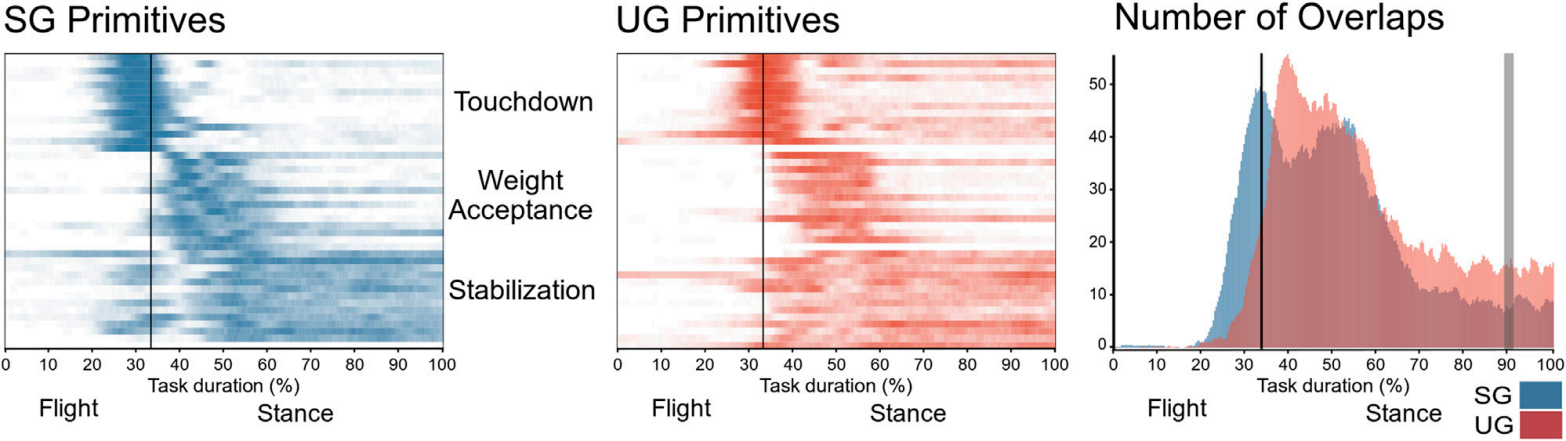
Overlapping time intervals of motor primitives for the single-leg drop landings on stable (SG, right panel blue) and unstable ground (UG, middle panel red). Each row of the heat maps represents a single motor primitive. A colored time point indicates the primitive is exceeding half maximum. Darker colors indicate higher number of occurrences across all cycles per participant. At the right panel, the average number of overlaps across all trials and all participants per ground condition with gray bands denoting time period of significant difference found by the statistical parametric mapping. For all graphs, the *x*-axis full scale represents one trial time-normalized to 300 points. The vertical line indicates the touchdown.

The number of extracted synergies that sufficiently reconstructed the original EMG signals did not differ between the two ground conditions (SG = 4.64 ± 0.49, UG = 4.85 ± 0.53, *p* = 0.282). We identified three fundamental synergies on both SG and UG (Figure 5). The first synergy was functionally related to the preparation of touchdown and showed a major contribution of plantar flexors. The second synergy presented its main activity shortly after the touchdown, thus it was functionally related to the weight acceptance and showed a main contribution of knee extensors. The third synergy represented the stabilization phase after landing and was characterized, in SG, by a major contribution of the muscles acting around the ankle joint, while in UG we observed a main contribution of hamstrings, tibialis anterior, and peroneus longus. A significant interaction of ground by muscle was observed in the motor module of the touchdown synergy (F(12, 144) = 2.594, *p* = 0.004). The *post hoc* analysis showed a higher contribution of gluteus medius (*p* = 0.015) and a lower contribution of gastrocnemius lateralis (*p* < 0.001) when landing on UG compared with SG (Figure 5). An interaction of ground by joint (F(2, 24) = 6.347, *p* = 0.006) was observed in the CaI of muscles in the touchdown synergy. The *post hoc* analysis showed that landing on UG significantly increased coactivation around the knee joint compared with SG (*p* = 0.001, Figure 6). The FWHM of the touchdown primitive was in UG on average 61 ± 17 points and was significantly greater (F(1,13) = 11.27, *p* = 0.005) than in SG (48 ± 7 points). The overlaps of the motor primitives showed a statistically significant difference (t* = 4.752, *p* < 0.049) only at about 90% of the task duration (Figure 7).

## DISCUSSION

We investigated the effects of perturbations induced by unstable surfaces on the mechanical loading and modular organization of leg muscles during single-leg landings. We hypothesized a modulation of the neuromotor control when landing on UG resulting in an increase of leg muscle loading. When landing on UG, the participants modulated the spatiotemporal structure of muscle synergies mainly in the touchdown phase, indicating a proactive adjustment to the unstable surface and confirming our first hypothesis. The experience-based proactive control in combination with the deformation characteristic of the soft surface resulted in a lower maximum resultant ankle and hip joint moment, lower rate of joint moment development, and no increase in muscle EMG activity observed during the landing phase. Thus, the hypothesis of an increased muscle loading was rejected. Our results show that the participants managed to use their experience and awareness of the unstable ground characteristics to proactively deal with the predicted perturbation before touchdown, minimizing the consequences of the perturbation.

The modulation of the spatiotemporal structure of the touchdown synergy (i.e., widening of the motor primitive and modified contribution of gluteus medius and gastrocnemius medialis muscles) indicates proactive adjustments in the neuromotor control of landing on UG. Proactive control strategies have been shown to be very effective to support stability in the presence of perturbations and to prevent a fall (Patla, 2003; Bierbaum et al., 2010; Bohm et al., 2012). Moreover, proactive adjustments have been proposed to successfully compensate proprioceptive impairments (Gordon et al., 2020) and enhance passive stabilizing mechanisms (Moritz and Farley, 2004; Morey-Klapsing et al., 2007). In our experiment, the landings were performed with open eyes and participants had previously acquired knowledge about the ground and task characteristics during the familiarization trials. Therefore, it is likely that the spatiotemporal modifications found in the touchdown synergy reflect a proactive strategy driving the preparation to the predictable perturbation. Widening of motor primitives is a phenomenon commonly associated with the presence of perturbations which has been proposed to reflect a mechanism that increases the robustness of neuromotor control (Martino et al., 2015; Santuz et al., 2018; Munoz-Martel et al., 2019; Janshen et al., 2020). The reduced CoP area when landing on UG indicates that the proactive control successfully predicted most of the challenges induced by the compliant surface, facilitating landing stability (Morey-Klapsing et al., 2007).

**FIGURE 8:**
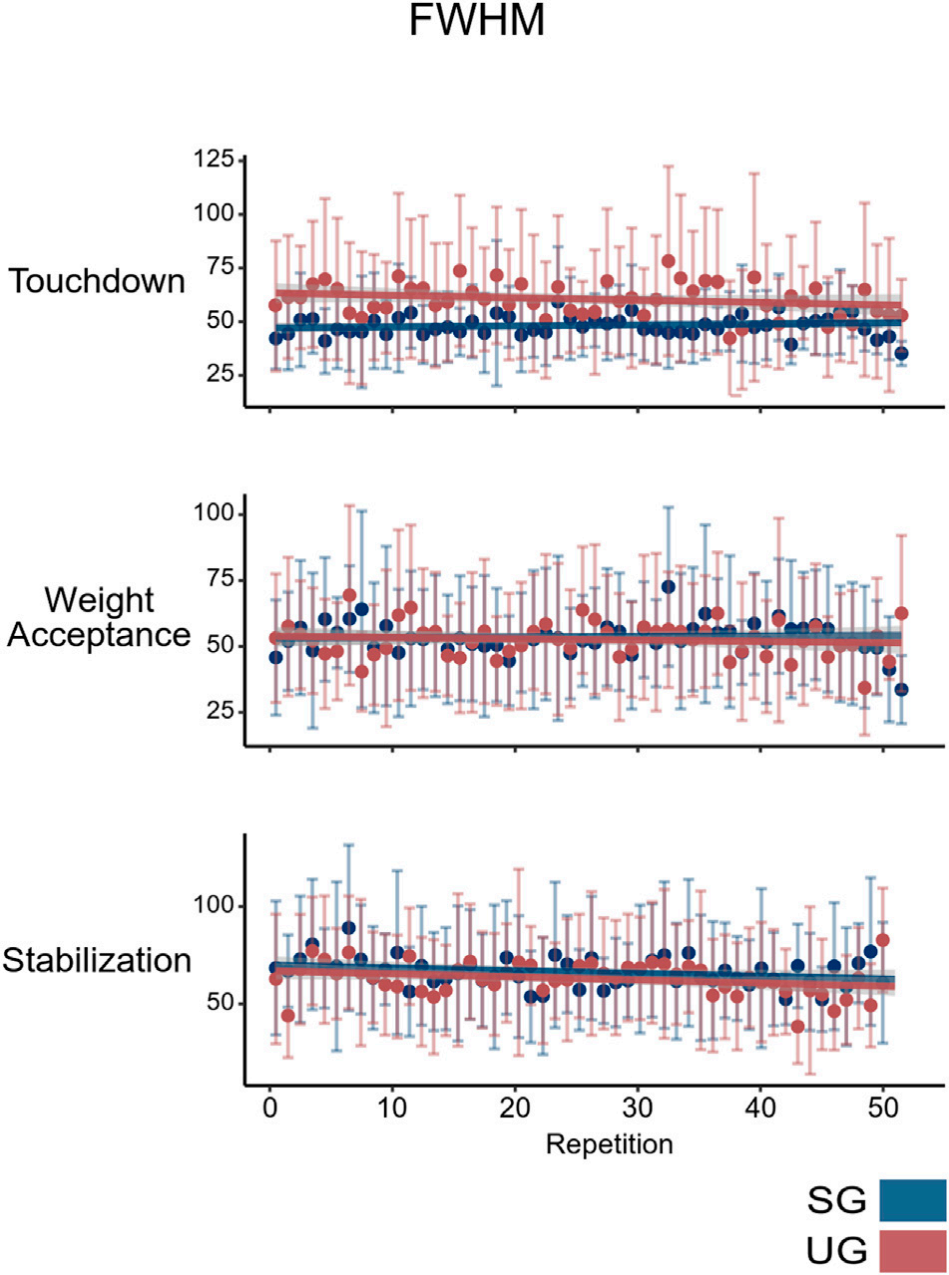
Mean value and SD of full width at half maximum (FWHM) of the classified motor primitives for every single-leg landing on stable (SG) and unstable (UG) ground. Lines represent the linear interpolation of each data set.

It is to mention that motor control can be quickly improved and the experience of just one or two trials in a predicted perturbation modifies significantly proactive strategies (Bierbaum et al., 2010; Bohm et al., 2012). In our statistical analysis, we used 50 landing trials in each condition and therefore the repeated experience on the unstable ground might introduce an acute, trial-dependent modification of the temporal structure of muscle synergies, potentially biasing the findings. To check for possible acute adaptations in the neuromotor control due to the repeated execution of the landings, we tested the FWHM of the motor primitives during the 50 repetitions using a linear mixed model. We did not find any effect of repetition on the FWHM of any of the three synergies: an indication that the basic activation patterns were not influenced by the landing repetitions (Figure 8). The participants performed some familiarization trials that were not included in this analysis. These initial repetitions might also have played a role in reinforcing previous knowledge of the landing characteristics initiating possible acute modifications in the modular organization and providing an adapted neuromotor control of the task.

When landing on UG, we observed a decreased CaI at the knee joint in the touchdown synergy indicating a higher contribution of the knee extensors compared with SG. Looking at the motor modules of the touchdown synergy, it is however visible that both knee flexors and extensors showed an almost negligible contribution to this synergy. Thus, the decreased CaI can be interpreted as functionally irrelevant. The knee joint plays a critical role during the landing phase to absorb the kinetic energy of the body (McNitt-Gray et al., 1993; Zhang et al., 2000; Hollville et al., 2020). The contribution of knee extensor muscles to the weight acceptance synergy is very high and the knee extension moment achieved its maximum in this phase, evidencing the importance of the knee joint for the kinetic energy dissipation during landings.

The weight acceptance and stabilization synergies were not modified in the UG condition and the overlapping of the motor primitives showed a short and small difference indicating a negligible influence of the unstable surface on the neuromotor control of the stance phase. Hence, it seems that predictive adjustment made by the participants during the single-leg landings were sufficient to cope with the UG and the unstable ground did not trigger reactive modulations of the neuromotor control which might be elicited if the difficulty of the task is increased. The result of our present setup is somewhat in disagreement with our previous findings during forward and backward lunging onto a foam beam—with similar mechanical characteristics to the current UG surface—where we found a modulation of the touchdown as well as the weight acceptance and stabilization synergies leading to a higher frequency of overlaps in the unstable condition (Munoz-Martel et al., 2021). From a biomechanical point of view, a basic difference between single-leg landings and lunges is the dynamic state of the body mass at touchdown. Landings were characterized by a vertical movement of the body center of mass with negligible components in the horizontal direction. On the other hand, the body mass moved in both horizontal and vertical direction during the forward and backward lunges. It seems that the two-dimensional body motion during the lunges was challenging to a greater degree the neuromotor control of the task in the presence of perturbations. This shows that the consequences of perturbations present a task specificity that should be accounted for when designing perturbation-based balance interventions. Sufficient reactive balance control after unpredicted perturbations is very important to maintain or even regain balance and avoid a fall. One of the main purposes of perturbation-based interventions is to improve balance reactive control, especially in older adults (Hamed et al., 2018a, 2018b; Mansfield et al., 2018; Gerards et al., 2021). Our results show that the unstable ground used for single-leg landings did not trigger reactive modulations of the neuromotor control and that predictive adjustment were sufficient to cope with the UG. Thus, we can argue that the use of unstable surfaces does not necessarily challenge reactive control. Challenging dynamic tasks (i.e., including anteroposterior and mediolateral body motion) or including a large catalogue of unstable conditions to increase the unpredictability of perturbations (Bohm et al., 2020) are key points in the design of perturbation-based interventions.

We expected an increase in the muscle activity and resultant joint moments as indicators of increased muscle loading in the UG condition. However, the ankle and hip maximum resultant joint moment and rate of moment development for all three joints were higher in SG. The damping behavior of the foam pads due to its viscoelastic properties might explain the significantly lower development of the GRF after touchdown and the reduced rate of joint moment development; the shorter lever arm of the GRF at the ankle joint, however, indicates an additional mechanism that explains the lower maximum ankle joint moment in UG. We found similar results (i.e., scarce differences in the EMG activity and a tendency toward lower resultant joint moments in the lower extremities) during forward and backward lunges on stable and unstable surfaces (Munoz-Martel et al., 2021). Therefore, we can conclude that using unstable surfaces does not necessarily increase muscle loading per se. We should remark that estimating resultant joint moments and the electromyographic activity of a muscle are indirect estimators of the mechanical demands for a muscle group. Nonetheless, both methods are valid and highly reliable and therefore provide an accurate estimation of the training stimuli*.* We should also remark that the foam pads used in the UG condition were bigger than the force plate and this might have transmitted a small portion of the landing forces to the ground. The size choice was dictated by the fact that pads as small as the force plate would show different mechanical properties and would lift their perimeter so strongly after landing that the foot would be completely enveloped and the effect of the foam strongly affected. Yet, our main focus was on the modular organization, thus we decided to use a bigger foam pad size, despite the potential bias in the measured GRF. In any case, we observed from the data that the vertical GRF at steady state was similar between SG and UG (i.e., body weight), indicating that the force dissipation due to the extra size might be negligible despite the acknowledged limitation.

In conclusion, our results provide evidence that the neuromotor system relied on a proactive control to modulate the spatiotemporal structure of muscle synergies during perturbed landing, particularly in the touchdown synergy. These modulations allowed the participants to deal with the predictable perturbation before touchdown and minimize the mechanical consequences of the perturbation. Moreover, our results show that the use of unstable surfaces did not challenge reactive motor control nor increase muscle loading per se. Since perturbation-based interventions aim to improve reactive balance, the task characteristics and the intensity of the challenge imposed by the unstable surface should be carefully designed when planning this kind of intervention programs.

## Data Availability

The raw data supporting the conclusion of this article will be made available by the authors, without undue reservation.
